# Comparison of the performance of the CMS Hierarchical Condition Category (CMS-HCC) risk adjuster with the charlson and elixhauser comorbidity measures in predicting mortality

**DOI:** 10.1186/1472-6963-10-245

**Published:** 2010-08-20

**Authors:** Pengxiang Li, Michelle M Kim, Jalpa A Doshi

**Affiliations:** 1 Division of General Internal Medicine, University of Pennsylvania School of Medicine, Philadelphia, Pennsylvania, USA; 2 Leonard Davis Institute of Health Economics, University of Pennsylvania, Philadelphia, Pennsylvania, USA; 3 Department of Health Care Management and Economics, Wharton School of Business, University of Pennsylvania, Philadelphia, Pennsylvania, USA

## Abstract

**Background:**

The Centers for Medicare and Medicaid Services (CMS) has implemented the CMS-Hierarchical Condition Category (CMS-HCC) model to risk adjust Medicare capitation payments. This study intends to assess the performance of the CMS-HCC risk adjustment method and to compare it to the Charlson and Elixhauser comorbidity measures in predicting in-hospital and six-month mortality in Medicare beneficiaries.

**Methods:**

The study used the 2005-2006 Chronic Condition Data Warehouse (CCW) 5% Medicare files. The primary study sample included all community-dwelling fee-for-service Medicare beneficiaries with a hospital admission between January 1^st^, 2006 and June 30^th^, 2006. Additionally, four disease-specific samples consisting of subgroups of patients with principal diagnoses of congestive heart failure (CHF), stroke, diabetes mellitus (DM), and acute myocardial infarction (AMI) were also selected. Four analytic files were generated for each sample by extracting inpatient and/or outpatient claims for each patient. Logistic regressions were used to compare the methods. Model performance was assessed using the c-statistic, the Akaike's information criterion (AIC), the Bayesian information criterion (BIC) and their 95% confidence intervals estimated using bootstrapping.

**Results:**

The CMS-HCC had statistically significant higher c-statistic and lower AIC and BIC values than the Charlson and Elixhauser methods in predicting in-hospital and six-month mortality across all samples in analytic files that included claims from the index hospitalization. Exclusion of claims for the index hospitalization generally led to drops in model performance across all methods with the highest drops for the CMS-HCC method. However, the CMS-HCC still performed as well or better than the other two methods.

**Conclusions:**

The CMS-HCC method demonstrated better performance relative to the Charlson and Elixhauser methods in predicting in-hospital and six-month mortality. The CMS-HCC model is preferred over the Charlson and Elixhauser methods if information about the patient's diagnoses prior to the index hospitalization is available and used to code the risk adjusters. However, caution should be exercised in studies evaluating inpatient processes of care and where data on pre-index admission diagnoses are unavailable.

## Background

Randomized controlled trials are not practically feasible in all situations given time, costs and/or ethical considerations. Observational studies using administrative claims data are being increasingly used in an attempt to address gaps in evidence where randomized trials are unavailable. However, the validity of the results from such secondary database analyses often face the threat of potential confounding associated with the differences in the baseline health status between patients. Risk adjustment methods are commonly used to address this threat with the most frequently used methods being the Charlson [1] and the Elixhauser [2] methods. These methods were developed for research purposes and utilize the International Classification of Diseases, Ninth Revision, Clinical Modification (ICD-9-CM) codes to determine a set of comorbidities shown to be predictive of outcomes including mortality.

The Centers for Medicare and Medicaid Services (CMS) has implemented a risk adjustment system, the CMS hierarchical condition categories (CMS-HCC) model to adjust capitation payments made to private plans in Medicare [3]. It "uses demographics and a diagnosis-based medical profile captured during all clinician encounters--both inpatient and outpatient--to produce a health-based measure of future medical need"[3,4]. In addition to generating a series of condition categories, it generates a summary risk score for each patient. Because it was originally developed for payment purposes, the CMS-HCC has been shown to be a significant predictor of health care costs, but has yet to be tested with health outcomes. Current methods of risk adjustment may be improved with the additional information retrieved by the CMS-HCC.

This study attempts to assess the performance of the CMS-HCC as a risk adjustment method in predicting mortality and to compare it with the Charlson and Elixhauser methods. We compare these three risk adjustment methods among all Medicare beneficiaries with a hospital admission and disease-specific samples consisting of patients with principal diagnoses of congestive heart failure (CHF), stroke, diabetes mellitus (DM), and acute myocardial infarction (AMI).

## Methods

### Data source

This study used data from the 2005 and 2006 Chronic Condition Data Warehouse (CCW) 5% Medicare files. Specifically, we used personal summary, inpatient, and outpatient files for a 5 percent random sample of Medicare beneficiaries. The inpatient file included one record per hospitalization summarizing all services rendered to a beneficiary from the time of hospital admission through discharge. The outpatient files included claims submitted by both institutional outpatient providers (e.g. hospital outpatient departments) and carrier claims submitted by non-institutional outpatient providers (e.g. physicians). We also used the personal summary file which captures demographic, enrollment, and administrative information on beneficiaries.

### Study samples

We evaluated the three risk adjustment methods in five samples. The primary study sample included all community-dwelling fee-for-service Medicare beneficiaries with a hospital admission between January 1^st^, 2006 and June 30^th^, 2006. Additionally, four disease-specific samples consisting of subgroups of patients who had a hospitalization with a primary diagnosis of (1) stroke (ICD-9-CM: '3623', '430', '431', '433'-'436'); (2) DM (ICD-9-CM: '250'); (3) AMI (ICD-9-CM: '410'); or (4) CHF (ICD-9-CM: '4254', '4255', '4257', '4258', '4259', '428') during the six-month observation period between January 1^st ^, 2006 and June 30^th^, 2006 were also selected. We selected the four conditions because they provide a mix of acute and chronic conditions that are common reasons for hospital admissions. Approximately 47% of all Medicare patients in the 5% CCW impatient file were discharged from hospitals during 2006 with at least one diagnosis of the four conditions. The first hospitalization during the six-month observation period was identified as the index hospitalization. The admission date of the index hospitalization was identified as the index date. Additional inclusion criteria for the study samples were fee-for-service Medicare Part A and B coverage for at least 12-month prior to the index admission and Medicare enrollment up to six months after the index date or death, whichever came first.

### Outcomes

The outcomes of interest were in-hospital all-cause mortality and six-month all-cause mortality. In-hospital mortality was defined as death during the index hospitalization stay. Six-month mortality was defined as death within 180 days of the index hospital admission. In-hospital mortality was also counted as part of the six-month mortality outcome. The vital status and date of death for each beneficiary was identified using the CCW personal summary file.

## Risk adjustment measures

### Charlson Method

The Charlson comorbidity index was originally derived to classify comorbidities that might alter the risk of mortality in individuals in longitudinal studies [1]. Nineteen comorbidities were identified with a relative risk of mortality of 1.2 or greater. A weighted index was created to take into account the number and seriousness of the comorbidities. There have been several adaptations of the Charlson index for use with administrative data. Each adaptation consists of a set of ICD-9-CM diagnosis codes associated with each of the comorbidities identified by the Charlson index. The Deyo [5] and Dartmouth-Manitoba adaptations [6] are the most common adaptations. It has been shown that only minor differences exist between the two methods and that they identify comparable numbers of comorbidities and have similar predictive power. In our study, the Charlson comorbidity measures were derived from the Dartmouth-Manitoba adaptation of the original Charlson method which includes broader definitions for peripheral vascular disease, complications of diabetes, and cancer. Nineteen comorbidity indicators were identified in this adaptation of the Charlson method [6].

### Elixhauser Method

The Elixhauser comorbidities method derives a comprehensive set of 30 comorbidities which were associated with increases in hospital length of stay, hospital charges, and in-hospital mortality [2]. The Elixhauser method extends the Charlson method by including additional comorbidities that were found to be strongly associated with these outcomes. Unlike the CMS-HCC and Charlson methods, the Elixhauser method does not provide a summary score. A weighted score was not derived because results showed that including the individual comorbidities were found to be more effective. However, for purposes of comparison with the other two risk-adjusters, we created a summary score for Elixhauser method by summing up the total number of Elixhauser comorbidities.

### CMS-HCC

The CMS-HCC model aggregates all ICD-9-CM codes into 189 condition categories which describe a broad set of similar diseases organized into organ systems [3]. Condition categories (CCs) are arranged in a hierarchy among similar diseases based on the severity of the disease which results in hierarchical condition categories (HCCs). Diagnoses were excluded if they were not medically significant, transitory or did not impact costs. A final count of 70 categories were developed to risk adjust costs. It then applies previously calibrated weights to each category to create a single risk score. The weights for each category were determined based on coefficients from a multiple regression on Medicare payments [3]. The traditional CMS-HCC risk adjustment method includes age, gender and Medicaid eligibility status for generating the CMS-HCC risk score. To ensure a fair comparison across the three risk adjustment methods, we removed the influence of these variables and only included the clinical condition categories to generate the CMS-HCC risk score. Also health services researchers often want to estimate the independent effects of demographic information (e.g. age, gender and eligibility status) while adjusting for the clinical disease burden. Thus, from a practical application standpoint it is useful to build the CMS-HCC risk score without patient demographic information.

### Analysis

Logistic regressions estimating mortality were used to compare the three risk adjustment methods, namely the Charlson, Elixhauser, and CMS-HCC method. A summary score version and clinical condition indicators version was created for each of these risk adjustment methods, thus resulting in a comparison of six risk-adjustment approaches. Programs to generate the CMS-HCC, Elixhauser, and Charlson risk scores were downloaded from the websites of CMS [7], Agency for Healthcare Research and Quality (AHRQ) [8], and Manitoba Center for Health Policy, University of Manitoba [9], respectively.

Traditionally, only inpatient files were used to code Charlson and Elixhauser comorbidities [1,2,5]. However, conditions from CMS-HCC were assigned not only using all diagnoses from inpatient files but also hospital outpatient and physician claims files [3]. More recently, outpatient and physician claims have also been used to code Charlson Comorbidities [10,11]. For a fair comparison of the different methods, we used the same analytic file to code each of the three risk adjustment methods. We created four analytic files for the primary sample and each of the four disease-specific samples (CHF, stroke, DM, and AMI). The first analytic file (IPindex) only included claims from the index hospitalization for each sample. The second file (IPpre12mo+index) included all inpatient claims in the 12 months prior to the index hospitalization including those occurring on the index admission date. The third analytic file (IP+OPpre12mo+index) included all inpatient and outpatient claims in the 12 months prior to the index hospitalization including those occurring on the index admission date. The fourth analytic sample (IP+OPpre12mo) included all inpatient and outpatient claims in the 12 months prior to the index hospitalization excluding those occurring on the index admission date.

Clinical diagnoses (e.g. comorbidities) under the three risk adjustment methods were identified using the ICD-9-CM diagnosis codes on the claims included in each of the four analytic files for the five samples. Summary scores and binary indicators of the clinical diagnoses were created to allow comparison of the six risk adjustment approaches in predicting the two outcomes, in-hospital mortality and six-month mortality. A total of 240 logistic regression models were estimated [5 samples*4 analytic files*6 risk adjustments methods (3 summary score and 3 separate indicators) *2 outcomes]. Each model included variables on the patient's age (as a linear and quadratic term) and gender.

Model performance was assessed using the c-statistic, the Akaike information criterion (AIC) [12], and the Bayesian information criterion (BIC) [13] in predicting mortality. The c-statistic is a measure of model discrimination and is equivalent to the area under the receiver operating characteristics (ROC) curve [14]. The c-statistic value of 0.5 indicates that the model's performance of prediction is equal to random chance. The highest value of c-statistic is 1 which indicates the perfect discrimination power of the model. Due to issues of over-fitting with the c-statistic, we used AIC and BIC as secondary measures for additional checks to determine goodness of fit. The AIC and BIC introduce a penalty term for additional parameters in the model, thereby reducing the problems associated with overfitting [15]. The AIC and BIC can be used for model comparison among a class of parametric models having the same dependent variable, but with different numbers of independent parameters. Within a set of estimated models, the models with the lowest AIC and BIC values are preferred. Unlike the F-test or the likelihood ratio test, the models being compared do not need to be nested [16]. We also computed the 95% confidence intervals for the c-statistic, AIC, and BIC using bootstrap methods. We generated 3000 bootstrap replicates of the original data set using random sampling with replacement. In each bootstrap sample, we ran all models to generate the c-statistic, AIC, and BIC.

## Results

The primary sample included a total of 170,342 patients with a hospital admission between January 1^st ^2006 and June 30^th ^2006. The mean age of the sample was 78 years and 40% were males. The inpatient mortality rate was 3% and six-month mortality rate was 14%. Across the four disease-specific samples a total of 2,339 DM, 9,828 stroke, 5,749 AMI, and 11,287 CHF patients were included in our study. The mean age of the patients ranged from 73.3 (DM) to 80.1 years (CHF) and the percentage of males ranged from 40.2% (stroke) to 49.6% (DM). Inpatient mortality was the highest for AMI (8.5%), followed by stroke (5.5%), CHF (3.6%), and DM (1.7%). Six-month mortality rates were similar and highest in CHF (23.3%) and AMI (23.1%) patients followed by stroke (18.1%) and DM (14.4%) patients.

Table 1 lists the c-statistic values from the logistic regression models estimating the risk of in-hospital mortality for patients in each of the five samples. In models using the summary score for risk adjustment, the CMS-HCC method achieved statistically significant higher levels of discrimination relative to the Charlson and Elixhauser methods for the primary sample and all four disease samples except for the fourth analytic file (IP+OPpre12mo) in DM patients. In models using the individual diagnosis indicators for risk adjustment, convergence was not complete for some models in disease-specific samples due to complete or quasi-complete separation [17]. C-statistic values were not available for these models and are not shown. Among models with c-statistic values available for all three risk adjusters, the CMS-HCC indicator models had significantly higher c-statistic values than the Charlson and Elixhauser indicators models.

**Table 1:** C-statistic from logistic regression models predicting in-hospital mortality^a^

Study sample	Analytic file used to code risk adjusters^b^	Summary score			Indicators		
		
		CMS-HCC	Charlson	Elixhauser	CMS-HCC	Charlson	Elixhauser
**All patients^c ^(N = 170,342)**							

	1) IPindex	0.853	0.649**	0.632**	0.917	0.68**	0.761**
		(0.849, 0.858)	(0.641, 0.656)	(0.624, 0.639)	(0.913, 0.921)	(0.673, 0.688)	(0.755, 0.768)
	2) IPpre12mo+index	0.826	0.66**	0.649**	0.904	0.682**	0.751**
		(0.821, 0.831)	(0.652, 0.668)	(0.642, 0.657)	(0.901, 0.909)	(0.675, 0.689)	(0.745, 0.758)
	3) IP+OPpre12mo +index	0.798	0.669**	0.67**	0.889	0.683**	0.735**
		(0.793, 0.803)	(0.661, 0.676)	(0.664, 0.678)	(0.886, 0.894)	(0.676, 0.691)	(0.729, 0.742)
	4) IP+OPpre12mo	0.665	0.652**	0.643**	0.693	0.662**	0.675**
		(0.658, 0.672)	(0.644, 0.659)	(0.636, 0.651)	(0.688, 0.701)	(0.655, 0.67)	(0.669, 0.683)
DM (N = 2,339)							
	1) IPindex	0.772	0.615**	0.627**	NA	0.674	NA
		(0.722, 0.853)	(0.546, 0.723)	(0.549, 0.726)		(0.634, 0.79)	
	2) IPpre12mo+index	0.764	0.664**	0.651**	NA	NA	NA
		(0.701, 0.835)	(0.599, 0.753)	(0.574, 0.744)			
	3) IP+OPpre12mo +index	0.744	0.674**	0.654**	NA	NA	NA
		(0.68, 0.816)	(0.62, 0.76)	(0.596, 0.746)			
	4) IP+OPpre12mo	0.662	0.69	0.644**	NA	0.73	NA
		(0.602, 0.751)	(0.618, 0.768)	(0.575, 0.729)		(0.694, 0.814)	
Stroke (N = 9,828)							
	1) IPindex	0.783	0.574**	0.575**	NA	0.607	0.672
		(0.759, 0.798)	(0.552, 0.6)	(0.553, 0.601)		(0.59, 0.636)	(0.657, 0.702)
	2) IPpre12mo+index	0.749	0.576**	0.581**	NA	0.62	0.663
		(0.722, 0.764)	(0.554, 0.603)	(0.557, 0.607)		(0.604, 0.65)	(0.649, 0.693)
	3) IP+OPpre12mo +index	0.72	0.577**	0.591**	NA	0.623	0.656
		(0.695, 0.736)	(0.555, 0.604)	(0.57, 0.618)		(0.609, 0.655)	(0.644, 0.689)
	4) IP+OPpre12mo	0.592	0.576**	0.578**	0.676	0.615**	0.614**
		(0.57, 0.618)	(0.553, 0.602)	(0.557, 0.606)	(0.675, 0.718)	(0.601, 0.647)	(0.607, 0.652)

AMI (N = 5,749)							

	1) IPindex	0.767	0.621**	0.625**	NA	0.646	NA
		(0.746, 0.784)	(0.599, 0.648)	(0.601, 0.651)		(0.627, 0.675)	
	2) IPpre12mo+index	0.743	0.628**	0.628**	0.852	0.647**	0.705**
		(0.721, 0.762)	(0.605, 0.655)	(0.606, 0.655)	(0.84, 0.874)	(0.63, 0.678)	(0.688, 0.731)
	3) IP+OPpre12mo +index	0.721	0.63**	0.645**	NA	0.647	0.697
		(0.7, 0.743)	(0.606, 0.656)	(0.622, 0.671)		(0.631, 0.68)	(0.685, 0.729)
	4) IP+OPpre12mo	0.64	0.629**	0.634**	NA	0.648	0.659
		(0.617, 0.668)	(0.606, 0.655)	(0.611, 0.661)		(0.632, 0.68)	(0.648, 0.696)
CHF(N = 11,287)							
	1) IPindex	0.738	0.615**	0.609**	0.822	0.652**	0.718**
		(0.715, 0.759)	(0.587, 0.639)	(0.582, 0.634)	(0.809, 0.848)	(0.631, 0.681)	(0.701, 0.748)
	2) IPpre12mo+index	0.717	0.64**	0.632**	0.805	0.662**	0.709**
		(0.697, 0.741)	(0.615, 0.664)	(0.611, 0.662)	(0.793, 0.833)	(0.643, 0.69)	(0.691, 0.737)
	3) IP+OPpre12mo +index	0.697	0.639**	0.646**	0.772	0.658**	0.7**
		(0.677, 0.724)	(0.617, 0.667)	(0.625, 0.676)	(0.767, 0.806)	(0.643, 0.691)	(0.686, 0.732)
	4) IP+OPpre12mo	0.643	0.635**	0.635**	0.686	0.657**	0.676**
		(0.622, 0.673)	(0.613, 0.662)	(0.615, 0.667)	(0.686, 0.732)	(0.64, 0.689)	(0.665, 0.712)

Note: **:The c-statistic is significantly different from the c-statistic of the CMS-HCC model at the 5% level.

NA: Models did not converge due to complete (or quasi-complete) separation.

^a ^95% confidence intervals of c-statistics are in parentheses.

^b^. Four analytic files were used to code risk adjusters: "IPindex" only included claims from the index hospitalization for each of the four disease samples. "IPpre12mo+index" included all inpatient claims in the 12 months prior to the index hospitalization including the index admission date. "IP+OPpre12mo+index" included all inpatient and outpatient claims in the 12 months prior to the index hospitalization including the index admission date. "IP+OPpre12mo" included all inpatient and outpatient claims in the 12 months prior to the index hospitalization excluding the index admission date.

^c^. Patients with any hospital admission between January 1^st^, 2006 and June 30^th^, 2006.

Table 2 shows the c-statistic values from the logistic regression models predicting the risk of six-month mortality. Similar to the findings for in-patient mortality, the summary score based on the CMS-HCC models had statistically significant levels of higher discrimination than the other two summary score based risk adjustment methods among all analytic files and across all samples. The models with individual diagnosis indicators converged in all analytic files for all samples except in two analytic files of the DM sample. Across all disease samples, the CMS-HCC indicator models had statistically significant higher c-statistic values than the Charlson and Elixhauser methods.

**Table 2:** C-statistic from logistic regression models predicting six-month mortality^a^

Sample	Analytic file used to code risk adjusters^b^	Summary score			Condition indicators		
		
		CMS-HCC	**Charlson**	**Elixhauser**	CMS-HCC	Charlson	Elixhauser
**All patients^c ^(N = 170,342)**							

	1) IPindex	0.799	0.728**	0.680**	0.827	0.743**	0.757**
		(0.796, 0.802)	(0.725, 0.731)	(0.677, 0.684)	(0.824, 0.830)	(0.740, 0.746)	(0.754, 0.760)
	2) IPpre12mo+index	0.790	0.739**	0.7**	0.825	0.753**	0.766**
		(0.787, 0.793)	(0.736, 0.742)	(0.696, 0.703)	(0.823, 0.828)	(0.749, 0.756)	(0.763, 0.769)
	3) IP+OPpre12mo +index	0.774	0.735**	0.719**	0.819	0.754**	0.777**
		(0.771, 0.777)	(0.732, 0.738)	(0.716, 0.722)	(0.816, 0.822)	(0.751, 0.757)	(0.774, 0.780)
	4) IP+OPpre12mo	0.716	0.711**	0.696**	0.745	0.726**	0.739**
		(0.712, 0.719)	(0.717, 0.714)	(0.692, 0.699)	(0.742, 0.748)	(0.722, 0.729)	(0.736, 0.743)
DM (N = 2,339)							
	1) IPindex	0.736	0.706**	0.685**	0.796	0.721**	0.753**
		(0.712, 0.763)	(0.676, 0.734)	(0.654, 0.712)	(0.786, 0.832)	(0.699, 0.753)	(0.736, 0.787)
	2) IPpre12mo+index	0.74	0.724**	0.7**	0.796	0.739**	0.758**
		(0.719, 0.773)	(0.701, 0.755)	(0.679, 0.736)	(0.795, 0.84)	(0.723, 0.777)	(0.749, 0.797)
	3) IP+OPpre12mo +index	0.733	0.72**	0.699**	NA	NA	0.762
		(0.712, 0.767)	(0.697, 0.749)	(0.678, 0.733)			(0.752, 0.799)
	4) IP+OPpre12mo	0.703	0.698**	0.682**	NA	0.722**	0.737**
		(0.681, 0.742)	(0.676, 0.731)	(0.66, 0.719)		(0.708, 0.761)	(0.729, 0.78)
Stroke (N = 9,828)							
	1) IPindex	0.79	0.667**	0.662**	0.842	0.682**	0.728**
		(0.776, 0.799)	(0.651, 0.679)	(0.647, 0.675)	(0.833, 0.853)	(0.668, 0.695)	(0.717, 0.742)
	2) IPpre12mo+index	0.774	0.683**	0.684**	0.841	0.701**	0.736**
		(0.76, 0.784)	(0.67, 0.696)	(0.672, 0.698)	(0.832, 0.853)	(0.691, 0.716)	(0.725, 0.75)
	3) IP+OPpre12mo +index	0.752	0.679**	0.693**	0.83	0.712**	0.739**
		(0.739, 0.763)	(0.665, 0.692)	(0.682, 0.708)	(0.821, 0.842)	(0.699, 0.725)	(0.729, 0.754)
	4) IP+OPpre12mo	0.688	0.675**	0.679**	0.718	0.706**	0.709**
		(0.677, 0.703)	(0.662, 0.689)	(0.668, 0.695)	(0.712, 0.737)	(0.693, 0.719)	(0.701, 0.726)

AMI (N = 5,749)							

	1) IPindex	0.777	0.692**	0.678**	0.811	0.704**	0.749**
		(0.763, 0.789)	(0.677, 0.707)	(0.661, 0.693)	(0.801, 0.826)	(0.69, 0.721)	(0.736, 0.764)
	2) IPpre12mo+index	0.776	0.71**	0.705**	0.814	0.719**	0.75**
		(0.763, 0.789)	(0.695, 0.725)	(0.691, 0.721)	(0.805, 0.83)	(0.706, 0.736)	(0.738, 0.767)
	3) IP+OPpre12mo +index	0.77	0.713**	0.727**	0.813	0.725**	0.763**
		(0.757, 0.783)	(0.699, 0.729)	(0.714, 0.742)	(0.805, 0.829)	(0.713, 0.743)	(0.752, 0.78)
	4) IP+OPpre12mo	0.72	0.707**	0.712**	0.741	0.717**	0.732**
		(0.706, 0.737)	(0.693, 0.723)	(0.698, 0.728)	(0.736, 0.764)	(0.705, 0.735)	(0.721, 0.751)
CHF(N = 11,287)							
	1) IPindex	0.685	0.653**	0.642**	0.719	0.669**	0.7**
		(0.675, 0.697)	(0.642, 0.665)	(0.631, 0.654)	(0.712, 0.733)	(0.659, 0.681)	(0.69, 0.712)
	2) IPpre12mo+index	0.694	0.671**	0.668**	0.725	0.681**	0.709**
		(0.685, 0.707)	(0.661, 0.684)	(0.658, 0.681)	(0.719, 0.739)	(0.673, 0.695)	(0.701, 0.723)
	3) IP+OPpre12mo +index	0.69	0.666**	0.671**	0.721	0.679**	0.702**
		(0.682, 0.704)	(0.656, 0.679)	(0.662, 0.685)	(0.715, 0.736)	(0.671, 0.693)	(0.695, 0.716)
	4) IP+OPpre12mo	0.674	0.663**	0.664**	0.698	0.675**	0.688**
		(0.665, 0.689)	(0.654, 0.677)	(0.655, 0.679)	(0.694, 0.715)	(0.667, 0.69)	(0.682, 0.704)

Note: **: The c-statistic is significantly different from the c-statistic of CMS-HCC model at the 5% level;

NA: Models did not converge due to complete (or quasi-complete) separation.

^a^. 95% confidence intervals of c-statistics are in parentheses.

^b^. Four analytic files were used to code risk adjusters: "IPindex" only included claims from the index hospitalization for each of the four disease samples. "IPpre12mo+index" included all inpatient claims in the 12 months prior to the index hospitalization including the index admission date. "IP+OPpre12mo+index" included all inpatient and outpatient claims in the 12 months prior to the index hospitalization including the index admission date. "IP+OPpre12mo " included all inpatient and outpatient claims in the 12 months prior to the index hospitalization excluding the index admission date.

^c^. Patients with any hospital admission between January 1^st^, 2006 and June 30^th^, 2006.

The results for the models predicting risk of in-patient and six-month mortality showed that the CMS-HCC method had substantially higher discrimination than the other two risk adjustment methods when we included the index admission claim in coding the risk adjusters. This was done in the first (IPindex), second (IPpre12mo+index) and third analytic (IP+OPpre12mo+index) files which contained the index admission. When we only used the pre-index inpatient and outpatient claims (i.e. the fourth analytic file: IP+OPpre12mo) to code risk adjusters, the CMS-HCC generally still had better discrimination than the other two methods overall. However, the difference was much smaller. In models including the individual diagnosis indicators of each risk adjustment method, the Elixhauser method generally outperformed the Charlson method in both in-patient mortality and six-month mortality models with higher c-statistic values. Because our objective was to compare the CMS-HCC method to the Elixhauser and Charlson method, we did not conduct statistical tests to compare the Elixhauser and the Charlson methods against each other.

Table 3 highlights the importance of including or excluding the index date claim in coding diagnoses and the summary score across the different risk adjustment approaches by measuring changes in the c-statistic. After excluding claims on the index date from all inpatient and outpatient claims within 12 months prior to the index admission, there were generally drops in model performance in predicting the risk of in-patient and six-month mortality. However, the drop in performance varied depending on the sample and risk adjustment method. The CMS-HCC had statistically significant drops in the c-statistic. Even though the Charlson and Elixhauser also experienced drops in performance, the reductions were smaller. In spite of this, the CMS-HCC still performed as well or better than the other two methods when we used only pre-index claims to code risk adjusters. In the CMS-HCC models, the influence of including the index date claim had a larger influence on c-statistic values in models predicting in-patient mortality than in the models predicting six-month mortality. In general, the stroke sample had larger drops in the c-statistic for the CMS-HCC model than other disease-specific samples.

**Table 3:** Change in c-statistics upon exclusion of claims occurring on the index admission date.^a^

		Summary score			Indicators		
		
Outcome	Sample	CMS-HCC	Charlson	Elixhauser	CMS-HCC	Charlson	Elixhauser
In-hospital	All patients^b^	-0.133**	-0.017**	-0.027**	-0.196**	-0.021**	-0.06**
	(N = 170,342)	(-0.139,-0.127)	(-0.019,-0.014)	(-0.03, -0.024)	(-0.203, -0.189)	(-0.025, -0.017)	(-0.065, -0.054)
	DM	-0.082**	0.016	-0.010	NA	NA	NA
	(N = 2,339)	(-0.113, -0.035)	(-0.023, 0.029)	(-0.045, 0.008)			
	Stroke	-0.128**	-0.001	-0.013**	NA	-0.008	-0.042
	(N = 9,828)	(-0.147, -0.1)	(-0.006, 0.002)	(-0.02, -0.005)		(-0.017, 0.001)	(-0.055, -0.02)
	AMI	-0.081**	-0.001	-0.011**	NA	0.001	-0.038
	(N = 5,749)	(-0.095, -0.063)	(-0.005, 0.003)	(-0.017, -0.005)		(-0.009, 0.009)	(-0.053, -0.018)
	CHF	-0.054**	-0.004	-0.011**	-0.086**	-0.001	-0.024**
	(N = 11,287)	(-0.066, -0.041)	(-0.011, 0.002)	(-0.018, -0.002)	(-0.096, -0.06)	(-0.012, 0.007)	(-0.035, -0.009)
Six-month	All patients^b^	-0.058**	-0.024**	-0.023**	-0.074**	-0.028**	-0.038**
	(N = 170,342)	(-0.06, -0.056)	(-0.026, -0.022)	(-0.025, -0.022)	(-0.076, -0.071)	(-0.03, -0.026)	(-0.04,-0.036)
	DM	-0.030**	-0.022**	-0.017**	-0.029**	NA	-0.025**
	(N = 2,339)	(-0.038, -0.018)	(-0.031, -0.008)	(-0.024, -0.008)	(-0.044, -0.012)		(-0.038, -0.007)
	Stroke	-0.064**	-0.004**	-0.014**	-0.112**	-0.006**	-0.030**
	(N = 9,828)	(-0.07, -0.053)	(-0.006, -0.001)	(-0.017, -0.01)	(-0.119, -0.096)	(-0.01, -0.002)	(-0.035, -0.022)
	AMI	-0.050**	-0.006**	-0.015**	-0.072**	-0.008**	-0.031**
	(N = 5,749)	(-0.057, -0.041)	(-0.009, -0.002)	(-0.02, -0.01)	(-0.08, -0.056)	(-0.012, -0.003)	(-0.038, -0.022)
	CHF	-0.016**	-0.003	-0.007**	-0.023**	-0.004	-0.014**
	(N = 11,287)	(-0.02, -0.012)	(-0.005, 0)	(-0.01, -0.003)	(-0.026, -0.016)	(-0.007, 0)	(-0.017, -0.007)

Note: **: The c-statistic of the model "IP+OPpre12mo+index" (included all inpatient and outpatient claims in the 12 months prior to the index hospitalization including the index admission date) is statistically significant different from the model "IP+OPpre12mo " (included all inpatient and outpatient claims in the 12 months prior to the index hospitalization excluding the index admission date) at the 5% level;

NA: Models did not converge due to complete (or quasi-complete) separation.

^a ^Change in c-statistics upon exclusion of claims occurring on the index admission date was estimated by comparing c-statistic of the model "IP+OPpre12mo+index" (included all inpatient and outpatient claims in the 12 months prior to the index hospitalization including the index admission date) and the c-statistic of the model "IP+OPpre12mo " (included all inpatient and outpatient claims in the 12 months prior to the index hospitalization excluding the index admission date); 95% confidence intervals of measures are in parentheses.

^b ^Patients with any hospital admission between January 1^st^, 2006 and June 30^th^, 2006.

The results of the AIC and BIC were consistent with those based on the c-statistic values (Data not shown. Results are available from the authors upon request). Overall, the CMS-HCC models performed better (i.e. had lower AIC and BIC values) than the Charlson and Elixhauser models in the analytic files across the five samples. However, some differences were not statistically significant. After excluding claims on the index date from all inpatient and outpatient claims within 12 months prior to the index admission, there were generally increases in the AICs and BICs.

## Discussion

Several studies have been conducted to compare the performance of the two most common risk adjustment methods, the Charlson and the Elixhauser methods [9,15,18,19]. This article adds to the literature by applying the CMS-HCC risk adjustment to predict mortality outcomes and comparing its performance with the Charlson and Elixhauser methods. Our findings suggest that the CMS-HCC method outperforms the Charlson and Elixhauser methods in predicting the risk of in-hospital and six-month mortality among all Medicare beneficiaries with a hospital admission including subgroups with a principal diagnosis of AMI, CHF, DM, and stroke. Based on point estimates of c-statistic, we also found that the Elixhauser method was superior to the Charlson method in models including individual diagnosis indicators to predict mortality. This is consistent with the previous literature [15,18,19].

There are several possible explanations for the superior performance of the CMS-HCC method. First, the CMS-HCC risk adjustment method captures more conditions than the Charlson and Elixhauser methods. The CMS-HCC aggregates 189 condition categories into 70 categories [3], while the Charlson identifies 19 comorbidities [1] and the Elixhauser identifies 30 comorbidities [2]. Second, the CMS-HCC has more detailed information on the severity of a condition relative to the Elixhauser and Charlson methods. For example, under the CMS-HCC method, a patient with diabetes can be coded as HCC19 (diabetes without complications), HCC18 (diabetes with ophthalmologic or unspecified manifestation), HCC17 (diabetes with acute complications), HCC16 (diabetes with neurologic or other specified manifestation), or HCC15 (diabetes with renal manifestation) [3] depending on the severity and complications associated with his/her diabetes. On the other hand, there are only two categories for diabetes under both the Charlson method (diabetes, mild to moderate vs. diabetes with chronic complications) [6] and the Elixhauser method (diabetes, uncomplicated vs. diabetes, complicated) [2]. Third, the CMS-HCC captures more complications that result from the process of care relative to the Charlson and Elixhauser methods. The Charlson and Elixhauser methods only include comorbidities and remove complications from the models given that one of the main purposes of these risk adjustment methods is to adjust for the baseline health status differences across patients before they were admitted to the hospital. Including complications due to the process of hospital care could overestimate severity of patient case mix among those who receive poorer inpatient quality of care. The CMS-HCC tries to capture all conditions associated with higher costs and hence includes complications because its original purpose was to predict Medicare expenditures. For example, pneumonia is included in CMS-HCC (as HCC111 and HCC112) but not in the Charlson and Elixhauser comorbidity lists because it is not distinguishable from a complication arising in the hospital [2]. Some diagnoses (e.g. myocardial infarction) included in the Charlson index might be due to complications of procedures (e.g. lumbar spine surgery) [5]. Therefore, the Charlson method includes them only if the condition occurred prior to the index hospitalization [5,6].

Inpatient mortality rates are often used to measure quality of care inside hospitals [20]. Risk adjustment is needed to ensure a fair comparison across hospitals by adjusting for patients' baseline clinical risk prior to hospital admission (e.g. comorbidities). However, if a risk adjustment method not only adjusts for pre-admission conditions, but also takes into account complications due to poor quality of inpatient care, it could potentially lead to a biased conclusion. The influence of complications due to the inpatient process of care can be eliminated by coding risk adjusters based on the pre-index date claims (i.e. the fourth analytical file in our study). However, as our results highlighted, removing the index date claim resulted in poorer predictions for both in-hospital and six-month mortality across all three methods. In-hospital mortality models had larger drops in c-statistic values than six-month mortality. The reason may be that in-hospital mortality is highly correlated with the conditions and complications during the inpatient stay. The CMS-HCC models had a larger drop in performance than the other two risk adjustment methods. In spite of this, the CMS-HCC still performed as well or better than the other two methods when we removed the influence of concurrent complications arising during the hospitalization by coding risk adjusters based only on the pre-index date claims.

In addition, the predictive power of the CMS-HCC models predicting in-hospital mortality decreased when additional diagnostic information from inpatient and outpatient claims in the 12-month prior to admission was included. This is in contrast to the Charlson and Elixhauser models whose performance in predicting in-hospital mortality increased with the inclusion of additional information. This difference may occur due to the fact that the clinical complications during the index hospitalization included only in the CMS-HCC model play an important role in its predictive performance for in-hospital mortality and adding prior diagnostic information dilutes its prediction power (Table 1 and Table 3).

The Charlson and Elixhauser methods were originally designed to serve as risk adjusters using only inpatient or hospital discharge data. Health services researchers have been increasingly using both inpatient and outpatient data for coding these two risk adjusters given the wide availability of longitudinal administrative claims datasets. However, these data also make it possible to exclude diagnoses codes related to complications due to inpatient processes of care by only including diagnoses from inpatient and outpatient claims identified before the index hospitalization date (i.e. exclude index hospitalization claim). In this case, our results indicate that the CMS-HCC is more favorable than the other two methods because it captures more comprehensive diagnosis information than the Charlson and Elixhauser methods and complications due to inpatient processes of care are not an issue. However, one should be careful when using CMS-HCC without removing diagnoses from index hospitalization particularly in studies evaluating inpatient processes of care.

There are several limitations of our study that should be addressed. First, some of our models using individual diagnosis indicators failed to converge. This may be due to our small sample sizes for some conditions which limited our ability to conduct a full comparison across all conditions and analytic files for the individual diagnosis indicator models across the three methods. However, all models converged for our primary sample of all patients with hospital admissions and the results were consistent with those from the models that converged in the disease-specific samples. Second, we limited our assessment of the outcomes to in-hospital and six-month mortality. Model performance may be different when examining a longer time horizon for mortality as well as for other health outcomes. Finally, our evaluation of the three risk-adjustment methods was conducted using samples of Medicare beneficiaries. Since the CMS-HCC risk score was originally developed and calibrated using data on Medicare beneficiaries whereas Charlson and Elixhauser methods were not, it is possible that our study results were more favorable for the CMS-HCC. However, one might argue that the CMS-HCC method was developed to predict Medicare expenditures and not mortality unlike the other two methods. Nevertheless, future evaluations of these risk adjustment methods in other patient populations are needed.

## Conclusion

The CMS-HCC risk adjustment models demonstrated better performance relative to the Charlson and Elixhauser models in predicting in-hospital and six-month mortality. The CMS-HCC model is preferred over the Charlson and Elixhauser methods if patient diagnoses prior to the index hospitalization are available and can be used to code these risk adjusters. However, it should be used cautiously in studies focused on evaluating inpatient processes of care when complications due to process of care cannot be identified and excluded based on available data.

The data used for the study is not openly available. This study is approved by University of Pennsylvania Institutional Review Board.

## Competing interests

The authors declare that they have no competing interests.

## Authors' contributions

PL participated in study design, data analysis and manuscript writing. MK and JD participated in study design and manuscript writing. All authors read and approved the final manuscript.

## Pre-publication history

The pre-publication history for this paper can be accessed here:

http://www.biomedcentral.com/1472-6963/10/245/prepub
